# MiR‐802 causes nephropathy by suppressing NF‐κB‐repressing factor in obese mice and human

**DOI:** 10.1111/jcmm.14193

**Published:** 2019-02-07

**Authors:** Da Sun, Jia Chen, Wei Wu, Ju Tang, Li Luo, Kun Zhang, Libo Jin, Sue Lin, Yitian Gao, Xiaoqing Yan, Chi Zhang

**Affiliations:** ^1^ Institute of Life Sciences, Wenzhou University Wenzhou China; ^2^ Zhejiang Province Engineering Laboratory for Pharmaceutical development of Growth Factors, Wenzhou Biomedical Collaborative Innovation Center Wenzhou China; ^3^ Sichuan Provincial Center for Mental Health, Sichuan Academy of Medical Sciences & Sichuan Provincial People's Hospital Chengdu China; ^4^ Bioengineering College, Chongqing University Chongqing China; ^5^ Medical Research Center, Southwest Hospital Third Military Medical University Chongqing China; ^6^ School of Pharmaceutical Sciences at the Wenzhou Medical University Wenzhou China; ^7^ The Third Affiliated Hospital of Wenzhou Medical University Ruian Wenzhou China

**Keywords:** inflammation, nephropathy, NF‐κB‐repressing factor, obesity

## Abstract

Obesity is associated with significant microvascular complications including renal injuries and may induce end‐stage renal disease. Emerging studies have demonstrated microRNAs (miRNAs) are potential mediators in the pathophysiological process of nephropathy. The present study aimed to investigate the role of miR‐802 in obesity‐related nephropathy and potential molecular mechanisms. Through utilizing obese mouse model and human subjects, we explored the therapeutic benefits and clinical application of miR‐802 in protecting against nephropathy. Renal miR‐802 level was positively correlated with functional parameters, including blood urea nitrogen and creatinine in obese mice. Specific silencing of renal miR‐802 improved high fat diet (HFD)‐induced renal dysfunction, structural disorders and fibrosis. The up‐regulated inflammatory response and infiltrated macrophages were also significantly decreased in miR‐802 inhibitor‐treated obese mice. Mechanistically, miR‐802 directly bond to 3ʹ‐UTR of NF‐κB‐repressing factor (NRF) and suppressed its expression. In clinical study, the circulating miR‐802 level was significantly increased in obese subjects, and positively correlated with plasma creatinine level but negatively correlated with creatinine clearance. Taken together, our findings provided evidence that miR‐802/NRF signalling was an important pathway in mediating obesity‐related nephropathy. It is a possible useful clinical approach of treating miR‐802 inhibitor to combat nephropathy.

## 1 INTRODUCTION

Concurrent with the worldwide obesity epidemic, there is a hugely increasing number of people with obesity‐associated metabolic diseases.^1^, ^2^ Obesity increases the risk of developing major risk factors for microvascular complications, like nephropathy and cardiomyopathy.^3^ Obesity‐related nephropathy is one of the key microvascular complications, which is characterized by increased glomerular filtration and blood urea nitrogen, structural disorders and dysfunctional in kidney.^4^ Eventually, it will develop into chronic kidney disease (CKD) and end‐stage renal disease (ESRD).^4^ However, the molecular changes of obesity‐related nephropathy are complicated. To protect against obesity‐associated nephropathy, it is urgent need to explore the underlying molecular mechanism.

Inflammatory response is the main phenotype of obesity‐related nephropathy. Excess nutrition, especially saturated fatty acids and its metabolites, injures the renal structure and function. Toxic‐free fatty acids can bind to toll‐like receptors (TLRs) resulting in the activation of IκB kinase/NF‐κB (IKK/NF‐κB) signalling pathways, which up‐regulate the synthesis and secretion of chemokines, leading to the infiltration of pro‐inflammatory immune cells.^5^, ^6^ Meanwhile, the obesity‐down‐regulated anti‐inflammatory signalling, such as NF‐E2‐related factor‐2 (Nrf2) and NF‐kappa‐B‐repressing factor (NRF), further exaggerates the inflammatory response.^7^, ^8^ Many findings also support renal inflammation mainly contributes to obesity‐associated nephropathy, whereas immunosuppressive strategies attenuate the development of diabetic nephropathy.^6^, ^9^, ^10^ Elevated circulating levels of inflammatory cytokines are positively correlated with renal injuries.^11^ Therefore, better controlling of inflammatory response is critical for suppressing the pathphysiological process of obesity‐related nephropathy.

MicroRNAs (miRNAs) are small non‐coding RNAs that are increasingly recognized as critical players in gene regulation and various diseases. Interestingly, a cluster of miRNAs is reported to be involved in the inflammatory process.^12^ Among them, recent studies show that several inflammation‐associated miRNAs are highly expressed in kidney. Renal glomerular miR‐192 regulates the process of diabetic nephropathy via inhibition of E‐box repressors.^13^ Renal miR‐146a positively correlates with the development of chronic renal inflammation and dysfunction.^14^ MiR‐21 promotes renal inflammation and fibrosis in diabetic mice.^15^ These findings support renal miRNAs are new promising biomarkers for diagnosis and therapeutic targets in nephropathy. MiR‐802, an emerging important mediator, is also increased in renal tissues of obese mice.^16^ The up‐regulation of inflammatory response also induces miR‐802 expression, and promotes cell proliferation in cholesteatoma.^17^ MiR‐802 participates in palmitate acid‐induced damage to pancreatic β cells through repression of sirtuin 6.^18^ Up‐regulation of miR‐802 induces apoptosis of keratinocytes in oral lichen planus.^19^ However, whether miR‐802 participates in the process of obesity‐associated nephropathy or the underlying molecular mechanism is still unclear.

Present study aimed to explore the potential role of miR‐802 in obesity‐associated nephropathy through high fat diet‐fed mouse model and human subjects. Here, we not only found a close correlation between miR‐802 and nephropathy parameters, but also suppression of miR‐802 could effectively improve cellular inflammatory response and renal function in obese mice. Mechanistically, *NRF*, an NF‐κB suppressor, was a potential direct target gene of miR‐802 in mediating renal disorders. Present study comprehensively provided the pathophysiological and clinical role of miR‐802 in obese‐associated nephropathy.

## 2 MATERIALS AND METHODS

### 2.1 Reagents

The biochemical assays for measuring blood urea nitrogen (BUN) and creatinine were purchased from Sigma chemicals (Sigma, St. Louis, USA). The lentivirus encoding miR‐802 sponge or control sponge was gifted from Dr Xiang Shen (Zhengzhou University). Haematoxylin, eosin solution, Sirius red staining kit were purchased from Sigma chemicals (Sigma, St. Louis, USA). Anti‐α‐SMA, anti‐collagen IV, anti‐CD68 and anti‐NRF antibodies were from Abcam (Cambridge, UK). Anti‐fibronectin antibody was from Sigma chemicals (Sigma, St. Louis, USA). Anti‐phosp‐IκB, anti‐IκB, anti‐p65, anti‐p50, anti‐Histone H1 and anti‐Tubulin antibodies were from Cell Signalling (Danvers, MA).

### 2.2 Animal experiment

Six‐week‐old male C57BL/6J mice were randomly assigned to normal chow (NC) or high fat diet (HFD, Cat#D12492, Research diets) for 16 weeks. Another set of animal study included obese mice fed high fat diet for 12 weeks were locally treated with 1 × 10^9^ lentivirus particles encoding miR‐802 sponge or control sponge by ultrasound‐microbubbles.^20^ Briefly, the lentivirus particle was mixed with Optison (Mallinckrodt, St. Louis, MO) in 50% v/v ratios, and injected into the renal artery. Ultrasound transducer (Sonitron 2000, NEPA GENE, Co.) exposed directly onto one side of the kidney with a continuous wave output of 1 MHz ultrasound for 1 minute. The infusion cannula is then removed, and the wound closed. These mice were fed HFD for another 4 weeks. Then, the mice were sacrificed, the serum and kidneys were collected for further analysis. The experimental procedure described here was approved by the Institutional Animal Use and Care Committee at the Wenzhou University (Wenzhou, China).

### 2.3 Renal histological analysis

Mouse kidneys were fixed in 4% paraformaldehyde for 24‐hour and embedded in paraffin. Paraffin sections of 5 µm were prepared and stained with haematoxylin and eosin solution or Sirius red staining kit. For immune histological for renal macrophages, slides were processed with antigen retrieval and 3% bovine serum albumin (BSA) blocking. Then, the slides were stained with anti‐CD68 antibody, secondary antibody and DAB HRP substrate for label‐positive stained cells. To measure the histological changes, the renal images were observed under a light microscope (400× amplification, Nikon, Tokyo).

### 2.4 Total RNA extraction, cDNA synthesis, reverse transcription and real‐time PCR

The total RNA was homogenized in TRIzol and isolated from mouse kidneys or human plasma according to the manufacturer's protocol. Reverse transcription was performed using the Superscript III Reverse Transcription System (Invitrogen) for mRNA or PrimeScript RT reagent Kit for miRNA, and real‐time PCR analysis was performed using SYBR Green or Taqman quantitative kit (Applied Biosystems, Alameda, CA). The sequence of primers for detecting mRNA was listed as following: TNF‐α: F‐5ʹ‐ACGGCATGGATCTCAAAGAC‐3ʹ; R‐5ʹ‐AGATAGCAAATCGGCTGACG‐3ʹ, IL‐6: F‐5ʹ‐GTCCTTCCTACCCCAATTTCCA‐3ʹ; R‐5ʹ‐TAACGCACTAGGTTTGCCGA‐3ʹ,iNOS: F‐5ʹ‐CCAAGCCCTCACCTACTTCC‐3ʹ; R‐5ʹ‐CTCTGAGGGCTGACACAAGG‐3ʹ, MCP‐1: F‐5ʹ‐CCACTCACCTGCTGCTACTCA‐3ʹ; R‐5ʹ‐TGGTGATCCTCTTGTAGCTCTCC‐3ʹ,NRF: F‐5ʹ‐AGAAAGATGGGTTGGACT‐3ʹ; R‐5ʹ‐CTGTGTGGCTCTCGGA‐3ʹ, GAPDH: F‐5ʹ‐AGGAGCGAGACCCCACTAAC‐3ʹ; R‐5ʹ‐GATGACCCTTTTGGCTCCAC‐3ʹ. Relative mRNA levels were normalized to GAPDH level. For miRNA expression analysis, 150 ng total RNA was reverse‐transcribed into cDNA using miRNA‐specific primers supplied with TaqMan MicroRNA Reverse Transcription kit. The quantitative real‐time PCR was performed using the ABI Prism 7000 instrument (Applied Biosystems). Small nucleolar RNA 202 (Sno202) was used as an internal control for comparison of relative changes in miRNA.

### 2.5 Western blot analysis

Protein extracts (50 μg) were run on 10% SDS‐PAGE. The protein was then transferred to a polyvinylidene difluoride membrane (PVDF, Amersham Biosciences). The membrane was blocked for 1‐hour at room temperature with 10% BSA in phosphate‐buffered saline/0.05% Tween 20. The blots were incubated overnight at 4°C with anti‐α‐SMA, anti‐collagen IV, anti‐fibronectin, anti‐phosp‐IκB, anti‐IκB, anti‐NRF, anti‐p65, anti‐p50, anti‐Histone H1 or anti‐Tubulin antibody and secondary antibody (Cell Signalling, Danvers, MA). The protein expression was visualized using enhanced chemiluminescence reagents (Bio‐Rad, Hercules, CA). The amounts of the proteins were analysed using Image J analysis software.

### 2.6 *NRF *gene 3ʹ UTR luciferase reporter assay

To generate *NRF* gene 3ʹ UTR luciferase reporter constructs, the miR‐802‐binding sites were synthesized by annealing the oligos: *NRF* 3ʹUTR forward: CTTCTTAATGCTTTCACCCCTCCGAACACACACCG; reverse: CTAATTGTGCAGGTACAGGAATTGTTCCACCAGCATTAATA. The products were ligated into the pMIR‐REPORT vector (Ambion). To create a mutant 3ʹ UTR, mutations were introduced at two miR‐802‐seeding sequence regions with the following sites: CA were changed to GC, and AGG were changed to GCC. HEK‐293T cells were transfected with one of the above plasmids using PEI (Polyplus) according to the manufacture's instruction. Luciferase activity was measured using the Dual‐Luciferase Reporter Assay (Promega). Data are presented as ratio of renilla to firefly luciferase activity.

### 2.7 Clinical study of human subjects

From October 2016 to December 2017, we have recruited 25 lean (BMI ≤ 23) and 20 obese (BMI > 28) individuals at the Sichuan Provincial People's Hospital. Exclusion criteria of this study included: individuals with known structural renal diseases, sepsis, electrolyte imbalance, chronic obstructive pulmonary disease, history of liver disease, malignancy, subclinical hyperthyroidism, history of drug abuse or pregnancy. Written informed consent was obtained from all participants and all the procedures were approved by human ethics committee of Sichuan Provincial People's Hospital.

### 2.8 Human anthropometric and biological measurement

The following data of all participants were collected from medical records: age, gender, personal medical history, family medical history, clinical manifestations, physical examinations, blood biochemical tests and echocardiograms. All blood tests were performed at the clinical laboratory of Sichuan Provincial People's Hospital. Blood biochemical tests included the levels of triglyceride, total cholesterol, high density lipoprotein‐cholesterol (HDL‐c), low density lipoprotein‐cholesterol (LDL‐c), fasting glucose and fasting insulin. Plasma insulin was measured by enzyme‐linked immunoassays (#90095, Crystal Chem, IL). Homeostasis Model of Assessment (HOMA) index was calculated to estimate insulin resistance (IR): HOMA‐IR = fasting glucose (mmol/l) × fasting insulin (mIU/l)/22.5. The plasma creatinine level was measured by automatic biochemical analyser (Hatachi, Japan). The Ccr was determined by injecting inulin into plasma, and the value was recorded in millilitres per minute of inulin excretion. For purification of cell‐free total RNA from human plasma, miRNeasy Serum/Plasma Kit (Qiagen, Cat#217184) was used for isolating miRs from 500 μL human plasma.

### 2.9 Statistical analysis

Data were presented as mean ± SEM. The Students’ *t* test was used for comparing two groups, and one‐way ANOVA was used for comparing four groups. GraphPad Prism 7 (GraphPad, San Diego, CA) was used to analyse the statistical significance between sets of data. Differences were considered to be significant at *P* < 0.05.

## 3 RESULTS

### 3.1 Elevated expression of renal miR‐802 is positively associated with obese mouse renal functional parameters

**Figure 1:**
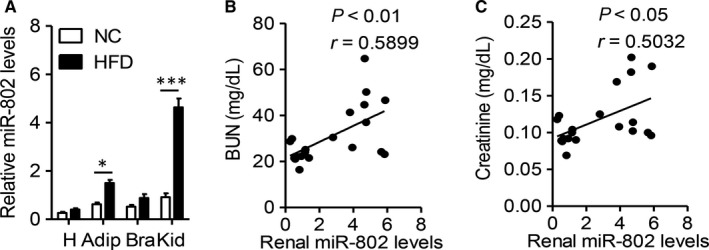
Renal miR‐802 level is closely correlated with renal function in obese mice. 6‐wk male C57BL/6J mice were fed normal chow (NC) or high fat diet (HFD) for 16 wk. A, Real‐time PCR analysis of miR‐802 levels in hearts (H), visceral adipose tissue (Adip), brain (Bra) and kidney (Kid). Significance was assessed by ANOVA test. (B‐C) Correlation between renal miR‐802 level and blood urea nitrogen (BUN, B) and serum creatinine (C). Correlation was assessed by non‐parametric Spearman's test. Data are shown as mean ± SEM (*P < 0.05 and ***P < 0.001, n = 8)

Previous study has showed miR‐802 participated in the development of metabolic diseases,^16^ but whether miR‐802 involved in renal pathophysiology is still unknown. First, we measured the expression of miR‐802 in different tissues, including heart, visceral adipose tissue, brain and kidney. As Figure 1A showed, high fat diet (HFD) obviously increased miR‐802 level in kidney (*P* < 0.001). Blood urea nitrogen (BUN) and creatinine are key markers for diagnosing nephropathy.^21^ We then measured serum levels of these two parameters and correlated with renal miR‐802 level. The increased level of miR‐802 was positively correlated with serum levels of BUN (Figure 1B) and creatinine (Figure 1C). These findings have explored the possible role of miR‐802 in regulating renal function in obese mice.

### 3.2 Renal miR‐802 inhibitor effectively improves high fat diet‐induced nephropathy

**Figure 2:**
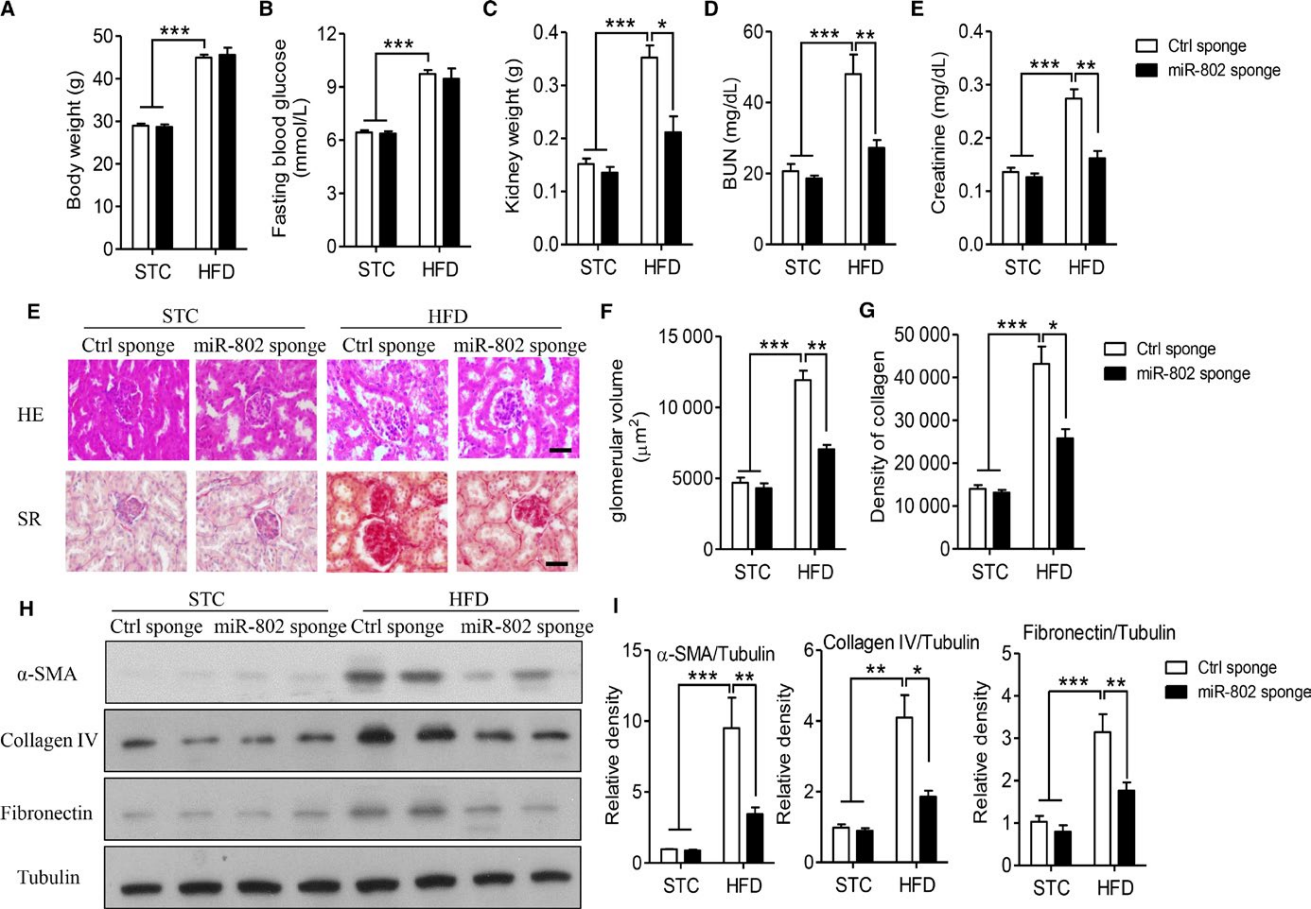
Blockage of renal miR‐802 protects against high fat diet‐induced renal dysfunction and structural disorders. Six‐week‐old male C57BL/6J mice were fed normal chow (NC) or high fat diet (HFD) for 12 wk. 1.2 × 10^9^ lentivirus particles encoding miR‐802 sponge or control sponge were delivered into renal tissue by ultrasound‐based microbubbles for 4‐wk. A‐C. Mouse body weight (A), fasting blood glucose level (B) and kidney weight (C). D‐E. Biochemical analysis of mouse BUN (D) and serum creatinine (E). E‐G. Haematoxylin & eosin (HE) and Sirius red (SR) staining of mouse kidney (E) and quantitative analysis of glomerular volume (F) and collagen deposits (G). Scale bar: 40 μm. H‐I. Western blot analysis of α‐SMA, collagen IV and fibronectin protein levels (H), and quantitative analysis of relative expression levels (I). Significance was assessed by ANOVA test. Data are shown as mean ± SEM (*P < 0.05, **P < 0.01 and ***P < 0.001, n = 6)

To determine the pathophysiological role of miR‐802 in diet‐induced renal injuries, we utilized ultrasound‐based microbubble carrying lentivirus delivery method to silence renal miR‐802 and investigated the changes of renal function and structure. The synthesized inhibitor, miR‐802 sponge, did not change the body weight (Figure 2A) and fasting blood glucose levels (Figure 2B), as compared with control (Ctrl) sponge treatment. However, miR‐802 sponge decreased the gain of kidney weight in obese mice (Figure 2C, *P* < 0.05). MiR‐802 sponge also effectively inhibited serum levels of BUN (Figure 2D, *P* < 0.01) and creatinine (Figure 2E, *P* < 0.001), as compared with Ctrl sponge‐treated obese mice. Abnormal glomerular enlargement and fibrosis are characters of nephropathy.^22^ The results of renal H&E staining supported the benefits of miR‐802 inhibitor in protecting against high fat diet‐induced structural disorders, including reduction of glomerular size and decreased thickness of basement membrane, as compared with ctrl sponge‐treated obese mice (Figure 2E,F). Besides, Sirius red staining showed suppression of renal miR‐802‐alleviated renal fibrosis (Figure 2E,G). Furthermore, western blot analysis also found miR‐802 sponge significantly inhibited the protein levels of fibrotic markers, including α‐SMA, collagen IV and fibronectin in obese mice (Figure 2H,I).

**Figure 3:**
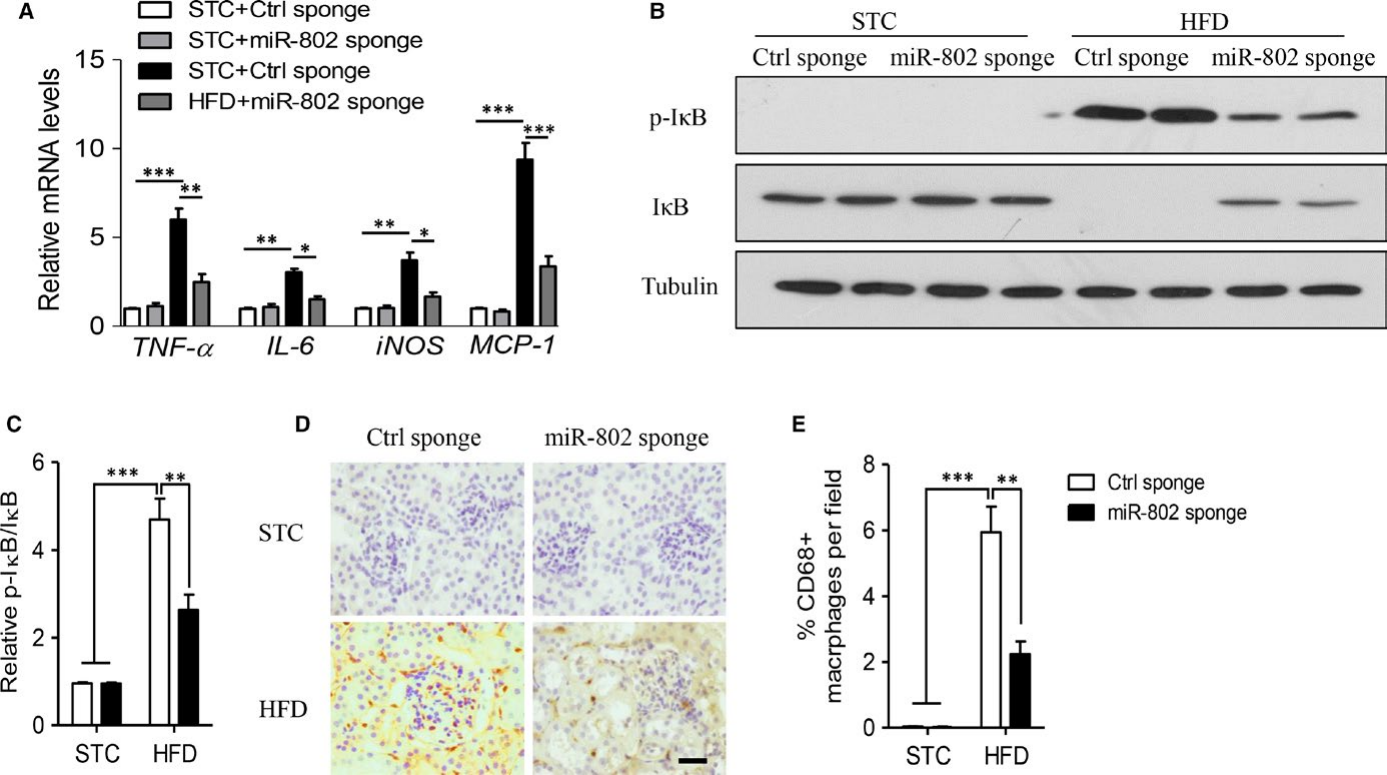
MiR‐802 inhibitor attenuates diet‐induced inflammation in mouse kidney. Six‐week‐old male C57BL/6J mice were fed normal chow (NC) or high fat diet (HFD) for 12 wk. 1.2 × 10^9^ lentivirus particles encoding miR‐802 sponge or control sponge were delivered into renal tissue by ultrasound‐based microbubbles for 4‐wk. A, Real‐time PCR analysis of mRNA levels of inflammatory cytokines. B‐C. Western blot analysis of phosphorylation (p)‐IκB and IκB in renal tissues (B), and quantitative analysis of relative protein density (C). D‐E. Immunohistological staining of macrophage marker CD68 in renal tissue section. Representative images of CD68^+^ cells (D) and quantitative analysis of relative percentage of CD68^+^ cells in total cell number (E). Significance was assessed by ANOVA test. Data are shown as mean ± SEM (*P < 0.05, **P < 0.01 and ***P < 0.001, n = 6)

Severe inflammatory response initiates and further deteriorates renal injuries in obese status.^11^ Real‐time PCR results showed that HFD obviously increased gene levels of several inflammatory factors, including *TNF‐α*, *IL‐6*, *iNOS* and *MCP‐1* (Figure 3A). Treatment of miR‐802 sponge effectively suppressed the mRNA levels of these inflammatory factors. NF‐κB signalling is the most important pathway to regulate inflammatory response in metabolic diseases.^5^, ^6^ As Figure 3B,C showed, miR‐802 sponge could inhibit the phosphorylation and degradation of IκB in obese mice, as compared with Ctrl sponge‐treated obese mice (*P* < 0.01). MiR‐802 sponge also decreased the translocation of p65 and p50 to nuclear, as compared with Ctrl sponge‐treated obese mice (Figure S1). The local inflammatory cytokines also recruit macrophage infiltration and amplify the inflammatory response.^11^ As showed Figure 3D,E, HFD stimulated CD68^+^ macrophage infiltration into renal tissues, whereas miR‐802 sponge significantly blocked macrophage accumulation (*P* < 0.01). However, local administration of miR‐802 sponge in kidney could not decrease the up‐regulation of circulating levels of inflammatory cytokines, including TNF‐α or IL‐1β in obese mice (Figure S2). Taken together, these results indicated that miR‐802 might mediate diet‐induced nephropathy through up‐regulating of renal inflammatory response.

### 3.3 MiR‐802 regulates renal inflammatory response through directly suppressing NF‐κB‐repressing factor

**Figure 4:**
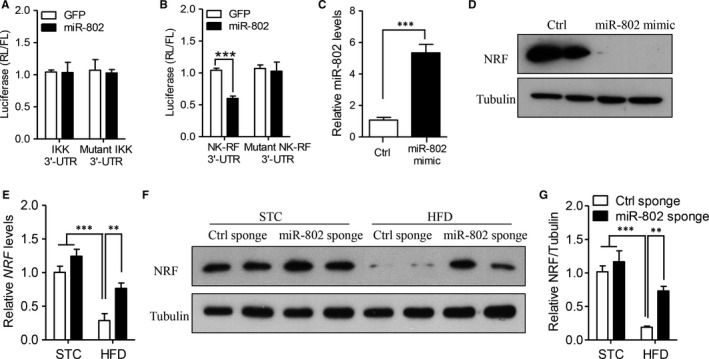
MiR‐802 directly suppresses gene expression of NF‐κB‐repressing factor (NRF). (A‐B) The dual‐luciferase reporter assay with plasmids encoding wild‐type *IKK* 3ʹ‐UTR (A), *NRF* 3ʹ‐UTR (B) or mutant 3ʹ‐UTR, transfected with miR‐802 overexpressed or control plasmid in HEK‐293 cells. C‐E. 5 × 10^5^ mouse mesangial cells were transfected with 1 × 10^6 ^IU lentivirus encoding miR‐802 mimic or control vector for 48 h. Real‐time PCR analysis of miR‐802 level (C) and western blot analysis of NRF (D) (n = 4 independent experiments). E‐G. Six‐week‐old male C57BL/6J mice were fed normal chow (NC) or high fat diet (HFD) for 12 wk. 1.2 × 10^9^ lentivirus particles encoding miR‐802 sponge or control sponge were delivered into renal tissue by ultrasound‐based microbubbles for 4‐wk. Real‐time PCR analysis of NRF mRNA level (E), western blot analysis of NRF expression in renal tissues (F) and quantitative analysis of relative protein density (G) (n = 6). Significance was assessed by ANOVA test (A‐B, E, G) and Students’ *t* test (C). Data are shown as mean ± SEM (**P < 0.01 and ***P < 0.001)

We next wanted to find out the direct target of miR‐802 in mediating inflammatory response. Previous studies have reported the activation of IκB is mainly controlled by IκB kinase (IKK) and NF‐κB‐repressing factor (NRF).^23^, ^24^ To this end, we utilized dual‐luciferase reporter assay to determine their interaction to miR‐802. Our showed that miR‐802 did not inhibit the luciferase density of IKK 3ʹ‐UTR (Figure 4A), but significantly suppressed the luciferase activity of NRF 3ʹ‐UTR (Figure 4B, *P* < 0.001). These findings indicated NRF was a direct target of mir‐802.

Furthermore, we overexpressed miR‐802 level in mouse mesangial cells (Figure 4C), and western blot analysis showed that overexpression of miR‐802 almost completely suppressed protein level of NRF (Figure 4D). In obese mice treated with miR‐802 sponge, miR‐802 inhibitor also significantly increased *NRF* gene level (Figure 4E) and protein level (Figure 4F,G). Above results strongly supported miR‐802‐induced renal inflammatory response and injuries through suppressing NRF in kidney.

### 3.4 Evaluated circulating miR‐802 is positively correlated with renal functional parameters in human subjects

To explore the clinical application of miR‐802 in diagnosing renal dysfunction in individuals, we collected plasma samples from 25 lean (BMI ≤ 23) and 20 obese (BMI > 28) human subjects. The clinical characteristics of the human subjects according to BMI categories were summarized in Table S1. As expected, obese subjects had significantly more adverse metabolic profiles including abnormal lipid profiles, hyperglycaemia and impaired insulin sensitivity.

**Figure 5:**
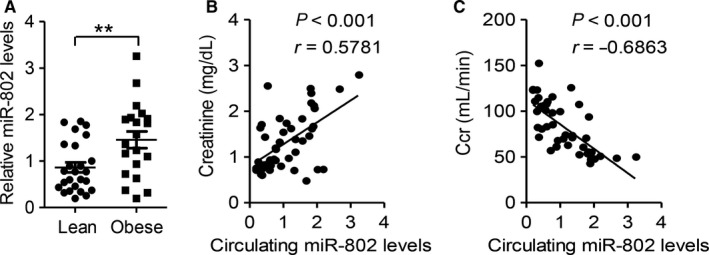
Circulating miR‐802 level is closely correlated with renal functional parameters in human subjects. Plasma from 25 lean (BMI ≤ 23) and 20 obese (BMI > 28) individuals were collected and subjected to analysis. A, Plasma level of miR‐802. Significance was assessed by Students’ *t* test. B‐C. Correlation between plasma miR‐802 level and creatinine (B) and creatinine clearance (Ccr, C). Correlation was assessed by non‐parametric Spearman's test. Data are shown as mean ± SEM (**P < 0.01)

Then, we measured and compared the circulating levels of miR‐802 in lean and obese subjects. As showed in Figure 5A, plasma from obese subjects had higher level of miR‐802 than from lean subjects (*P* < 0.01), which was consistent with mouse renal miR‐802 level. Then, we measured plasma creatinine levels and calculated the creatinine clearance (Ccr) in these subjects. As Figure 5B,C showed, plasma miR‐802 was positively correlated with creatinine levels (Figure 5B; *r* = 0.5781, *P* < 0.001), but negatively correlated with Ccr (Figure 5C; *r* = −0.6863, *P* < 0.001).

## 4 DISCUSSION

Increasing evidence shows that obesity‐induced renal inflammation and its consequent structural disorders are a critical process leading to end‐stage of nephropathy. Present study demonstrated that suppression of renal miR‐802 protected against the progressive renal injury through down‐regulation of renal inflammatory response in obese mice. Mechanistically, our findings also supported the protective role of miR‐802 inhibitor on nephropathy might be associated with the suppressive effect NF‐κB activity by its suppressing factor NRF. In human study, we also found the levels of miR‐802 were closely correlated with clinical parameters of nephropathy.

The pathogenesis of obesity‐related complications is characterized by activation of complex molecular pathways, and emerging evidence now suggests that inflammatory pathways have a central role in the development of these diseases, such as obesity‐related nephropathy. One of the major signalling pathways implicated in this inflammatory reaction is nuclear factor‐κB (NF‐κB), a transcription factor that is activated by many stimuli relevant to obesity‐related nephropathy, such as pro‐inflammatory cytokines and mechanical forces. In turn, activation of NF‐κB stimulates the synthesis and production of inflammatory molecules.^11^ Inhibition of tumour necrosis factor (TNF)‐α improved markers of glomerular and tubulointerstitial injury with diabetic nephropathy.^25^ Abnormalities of interleukin (IL)‐6 expression altered glomerular endothelial cell permeability, mesangial cell proliferation and expression of fibronectin.^26^ Local infiltration of immune cells, especially macrophages, exacerbated inflammatory response in the process of nephropathy.^11^ Mechanistically, the accumulated macrophages and inflammatory cytokines damaged endothelial cell permeability, metabolic homeostasis and structure. Consistently, present study also found the abnormal inflammatory activation in obese mice, but suppression of miR‐802 effectively improved high fat diet‐induced renal dysfunction in mice.

Nuclear factor (NF)‐κB is activated by IκB kinase (IKK)‐mediated phosphorylation of IκB, which triggers proteasomal IκB degradation. This enables the active subunits of NF‐κB to translocate to the nucleus and induce target gene expression. Suppression of NF‐κB transcriptional activity effectively inhibits the expression of downstream inflammatory and oxidative cytokines. Therefore, target on NF‐κB is a promising approach to protect against inflammation‐induced diseases. Selective inhibitor of IκB degradation or NF‐κB translocation, such as BAY 11‐7082 and JSH‐23, effectively suppressed its transcriptional activities.^27^, ^28^ These inhibitors could improve NF‐κB‐mediated inflammation and tissue damages in mouse and human studies.^27^, ^28^ In 1999, Nourbakhsh et al described the functional characterization of NRF (NF‐κB‐repressing factor), which abolishes the transcriptional activity of the bordering NF‐κB binding sites.^24^ Then, lots of studies continued to explore the biological roles of NRF in different molecular signalling. NRF participated in basal repression of pro‐inflammatory cytokine IL‐1‐induced activation of IL‐8.^29^ NRF was a negative mediator of inducible nitric‐oxide synthase (iNOS) in human A549 and HeLa cells.^8^ In severe COPD patients, there existed significant reduction of NRF expression, but enhanced NF‐κB activation.^30^ Consistently, present study also found the renal level of NRF was obviously decreased, but the inflammatory response was increased in obese mice. Luciferase assay also determined 3ʹ‐UTR of *NRF* was a direct binding site for miR‐802, which firstly provided the links between miRNAs and NRF.

A cluster of studies have reported miR‐802 was involved in the tissue dysfunction. MiR‐802 was identified as an important mediator in controlling different pathological process. Intestinal miR‐802 affected the biological efficacy of angiotensin II in gastrointestinal system.^31^ In obesity, up‐regulated hepatic miR‐802 impaired glucose metabolism through silencing of *Hnf1b*.^16^ In type 2 diabetic patients, the circulating miR‐802 was also significantly increased and closely correlated with metabolic parameters.^32^ These studies also addressed miR‐802 and inflammatory response had a positive feedback in the development of tissue dysfunction. Furthermore, a multitude of miRNAs have also been found to be associated with the process of renal diseases. The down‐regulation of circulating miR‐130b might be involved in the pathological development of diabetic nephropathy through inducing lipid metabolic disorders, oxidative stress, extracellular matrix deposition and renal fibrosis.^33^ Circulating miR‐217 was positively associated with the levels of proteinuria in type 2 diabetes patients.^34^ Besides, one study also identified several urea miRNAs had the capability of significantly differentiating patients with acute kidney disease (AKI) from individuals without AKI.^35^ All these findings supported miRNA is a useful clinical biomarker of nephropathy. More importantly, renal miR‐802 could mediate potassium channel activity through suppressing caveolin‐1 expression.^36^ In present study, we found up‐regulation of miR‐802 closely associated with obesity‐induced renal dysfunction, including structural disorders, fibrosis and accumulated inflammatory response. Furthermore, we firstly demonstrated that serum levels of miR‐802 were significantly increased in obese subjects with renal dysfunction.

However, there were still several limitations in present study. Due to multiple targets of miRNAs, it cannot exclude other mediators in the complicated pathology of nephropathy. But present study at least demonstrated miR‐802/NK‐FR signalling was one of the molecular mechanisms to explain the progress of obesity‐related nephropathy. Another unresolved issue was present study measured circulating miR‐802 level instead of local renal expression. As showed in Figure 1, renal tissue had higher level of miR‐802 in comparison with other tissues. However, it was more feasible to collect serum samples in clinical study. Further, we will measure the renal miR‐802 levels in clinical study after getting the approval from ethic committee.

In conclusions, present study unveiled a novel pathophysiological role of miR‐802 in the development of obesity‐related nephropathy through obese mice and human study. The therapeutic benefits of miR‐802 inhibitor and clinical correlation support miR‐802 are a potential biomarker in diagnosing renal dysfunction in obese subjects.

## CONFLICT OF INTEREST

The authors declare that there are no conflicts of interest.
