# Supplementary figures and images for: MiR‐802 causes nephropathy by suppressing NF‐κB‐repressing factor in obese mice and human
Source: J Cell Mol Med. 2019 Feb 7;23(4):2863–71. doi: 10.1111/jcmm.14193 (PMC6433720; doi:10.1111/jcmm.14193)

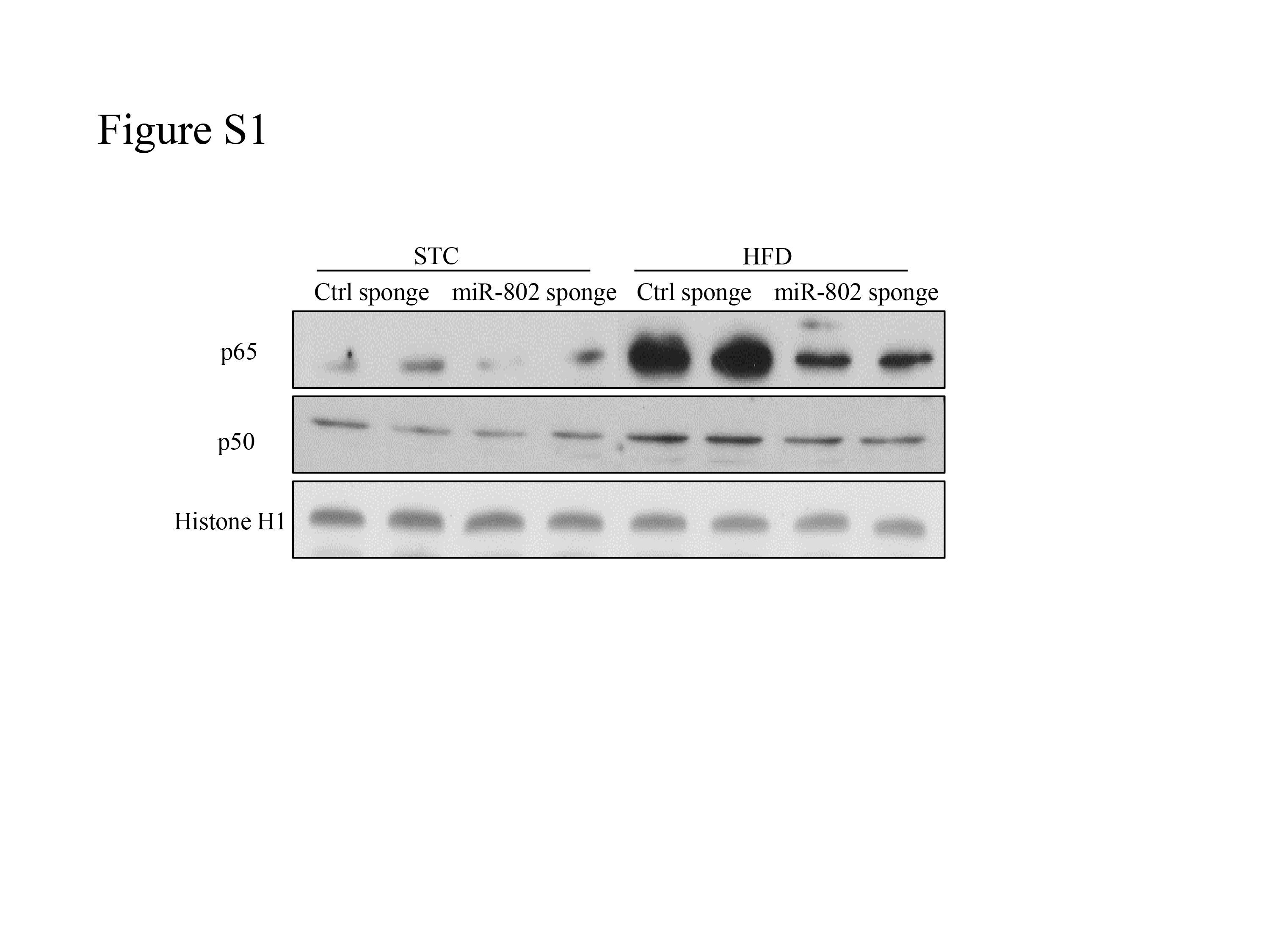

Supplement: Supplementary file 1 [file JCMM-23-2863-s001.tif]

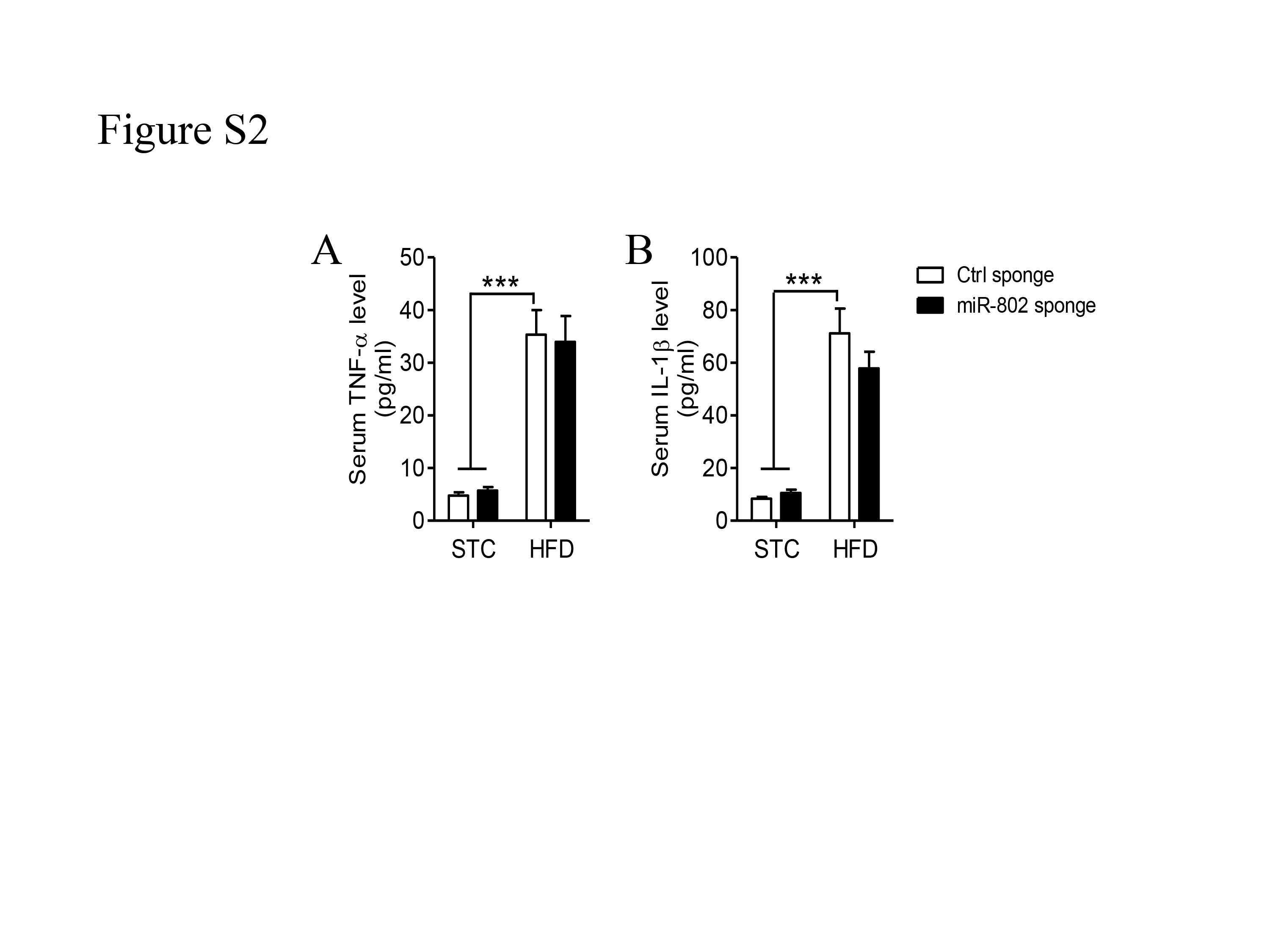

Supplement: Supplementary file 2 [file JCMM-23-2863-s002.tif]

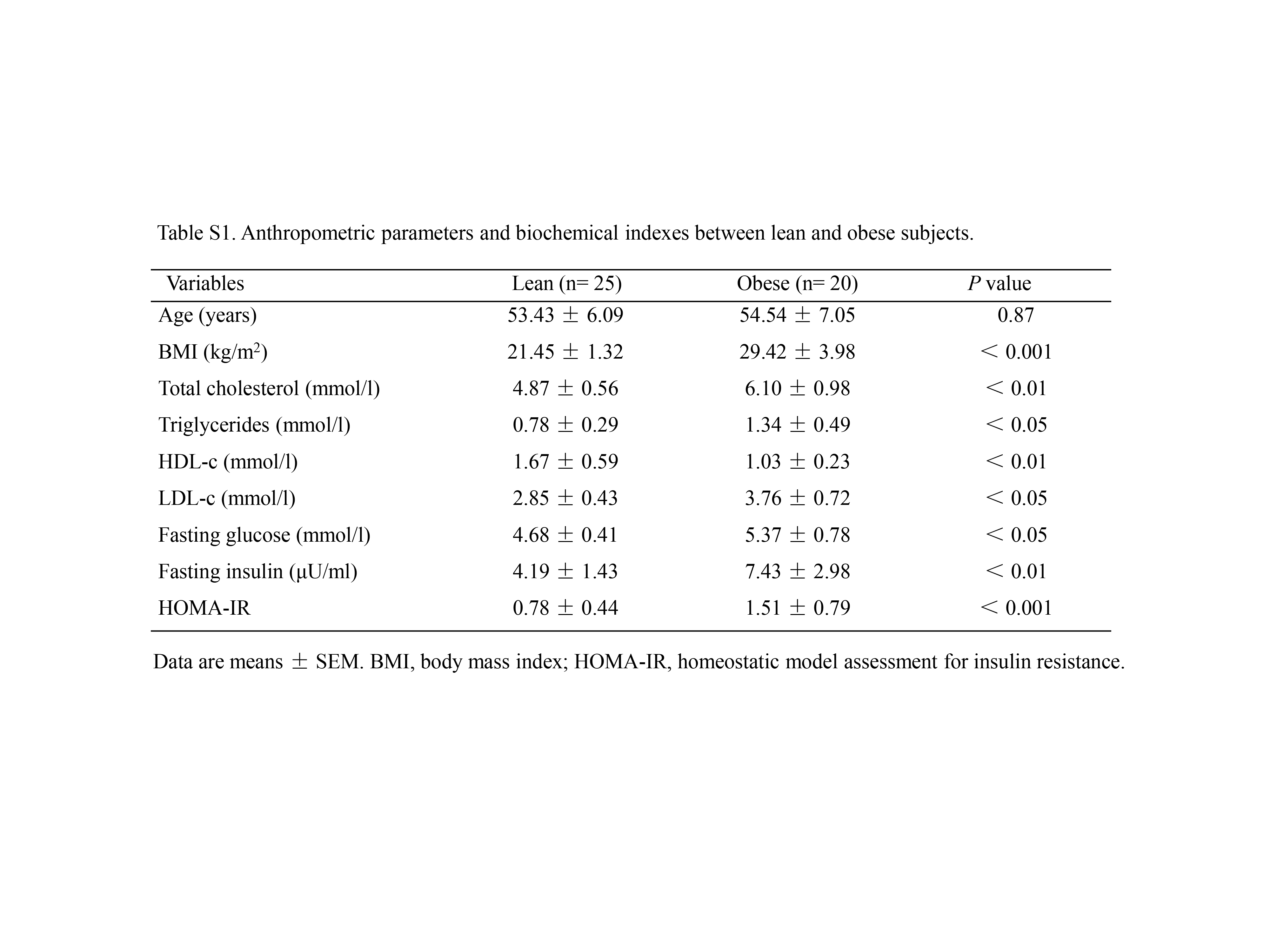

Supplement: Supplementary file 3 [file JCMM-23-2863-s003.tif]
