# Supplementary material for: MiR‐802 causes nephropathy by suppressing NF‐κB‐repressing factor in obese mice and human
Source: J Cell Mol Med. 2019 Feb 7;23(4):2863–71. doi: 10.1111/jcmm.14193 (PMC6433720; doi:10.1111/jcmm.14193)
Supplement: Supplementary file 4 [file JCMM-23-2863-s004.docx]

**Figure S1.** The protein expression of p65 and p50 in miR-802 sponge-treated obese mice. Six-week-old male C57BL/6J mice were fed normal chow (NC) or high fat diet (HFD) for 12 weeks. 1.2 $\times$ 10^9^ lentivirus particles encoding miR-802 sponge or control sponge were delivered into renal tissue by ultrasound-based microbubbles for 4 week. Western blot analysis of NF-κB p65 and p50 in extracted nuclear protein of renal tissues. Histone H1 was nuclear loading control.

**Figure S2**. The circulating levels of inflammatory cytokines in miR-802 sponge-treated obese mice. ELISA analysis of circulating levels of TNF-α and IL-1β. Significance was assessed by ANOVA test. Data are shown as mean ± SEM (*** p<0.001).
